# Effect of Transcriptional Regulator ID3 on Pulmonary Arterial Hypertension and Hereditary Hemorrhagic Telangiectasia

**DOI:** 10.1155/2019/2123906

**Published:** 2019-07-11

**Authors:** Vincent Avecilla

**Affiliations:** ^1^Department of Environmental Health Sciences, Robert Stempel College of Public Health & Social Work, Florida International University, Miami, FL 33199, USA; ^2^Celgene Corporation, Summit, NJ 07901, USA

## Abstract

Pulmonary arterial hypertension (PAH) can be discovered in patients who have a loss of function mutation of activin A receptor-like type 1 (*ACVRL1*) gene, a bone morphogenetic protein (*BMP*) type 1 receptor. Additionally,* ACVRL1* mutations can lead to hereditary hemorrhagic telangiectasia (HHT), also known as Rendu-Osler-Weber disease, an autosomal dominant inherited disease that results in mucocutaneous telangiectasia and arteriovenous malformations (AVMs). Transcriptional regulator Inhibitor of DNA-Binding/Differentiation-3 (*ID3*) has been demonstrated to be involved in both PAH and HTT; however, the role of its overlapping molecular mechanistic effects has yet to be seen. This review will focus on the existing understanding of how* ID3* may contribute to molecular involvement and perturbations thus altering both PAH and HHT outcomes. Improved understanding of how* ID3* mediates these pathways will likely provide knowledge in the inhibition and regulation of these diseases through targeted therapies.

## 1. Introduction

Inhibitor of DNA-Binding/Differentiation-3 (*ID3*) is part of the ID family of proteins (helix-loop-helix) programmed by an instant-early gene responsive to oxidative stress and mitogenic indicators. Known as a transcriptional regulator that prevents stem cell differentiation and promotes cell cycle progression,* ID3* has also been demonstrated to exhibit overlapping function as a gene knockout dependent on cellular context [1, 2]. With regard to the vasculature, ID3 is important in embryonic vasculogenesis as well as endothelial cell activation [1, 3, 4]. Given that these functions are observed in endothelial cells from diseased vasculature [5],* ID3* may mediate pulmonary dysfunction often found in individuals with cardiopulmonary disease such as pulmonary arterial hypertension (PAH), hereditary hemorrhagic telangiectasia (HHT), and atherosclerosis [6–10]. Furthermore,* ID3 *has been demonstrated to be a downstream target of the TGF*β*/BMP7 signaling pathway, which has been known to play a significant role in various cellular processes such as migration, apoptosis, and proliferation alongside the processes of angiogenesis using two pathways: ALK5-SMAD2/3 pathway and ALK1-SMAD1/5/8 pathway, important processes/pathways involved in PAH and HHT [11–14]. An enhanced understanding of interaction between ID3 and overlapping molecular mechanisms that can be mediated in PAH and HHT is essential in strengthening our complete understanding of how transcriptional regulators such as* ID3* can modify PAH and HHT outcomes. Additional study in these particular areas may show both more effective or innovative therapeutic modalities and provide strategies for the future.

## 2. Inhibitor of DNA-Binding/Differentiation-3 (ID3)

*ID3* is part of a family of proteins (*ID1*,* ID2*,* ID3*,* ID4*). The ID family shares a widespread amino acid homology within their domain (helix-loop-helix; 69-78%) [2, 15]. Studies have shown that in genetically engineered mice, the significance of* id3* in embryonic cell differentiation and development [16]. In dissimilarity,* id1*-*id3* knockout mice have demonstrated neuronal differentiation, irregular vascularization of the brain, and cardiac deficiencies [17, 18] that were embryonically fatal. Reports indicate that* ID3* is greatly expressed in embryonic tissue however decreases as cells differentiate [2]. The expression of* ID3* in adult tissues suggests a particular context and inclines to be at its peak in undifferentiated and proliferating cells.* ID3* expression has been described as being stimulated by different stimuli in various cell types [19].

*ID3* was originally recognized as a serum inducible instant-early gene in fibroblasts of mice that peak transcriptionally at 1 hour [20, 21].* ID3* expression consequently has also been described to be biphasic with leading stimulus at 1 hour following a secondary burst of 24 hour as in the event of tissue regeneration after injury.* ID3* protein-protein interaction occurs in mammals through the HLH transcriptional factors E proteins, which include HEB, E2-2, and E12/47.* TCF12*,* TCF3*, and* TCF4* genes encode these E proteins, respectively [22]. ID3 plays an essential function in cell proliferation through its connections with E proteins. E proteins have been demonstrated to bind the E-box sequence in the promoter p21^Cip1^ and trigger its transcription [23]. In perspective of the cell cycle,* ID3* stimulates cell cycle progression by the inhibition of p21^Cip1^ expression. Protein-protein interactions particularly with* ID3* and E proteins can disturb their capability of binding gene promoters and thus block transcriptional initiation by these factors [24]. Furthermore,* ID3* has been revealed to inhibit E proteins from stimulating the p21^Cip1^ promoter in proliferating vascular cells [25].

*ID3* has been indicated to control the binding of transcription factor 3 (TCF3) to the E-box motif in target gene promoters [26]. It has been reported that TCF3 represses the* SOX2*,* NANOG*, and* OCT4* expression that contributes to cell differentiation [20]. Previous research has displayed overexpression, which increased both* SOX2* and* OCT4* expression in endothelial cells. This resulted in a population of cells that were positive for molecular stemness signature CD133+ VEGFR3+ CD34+ [27]. These endothelial stem cells were differentiated into neuron and smooth muscle cells. Based on this information,* ID3* maintains cells in a noncommittal state by counteracting the repression of pluripotency factors by TCF3.

### 2.1. Pulmonary Arterial Hypertension (PAH)

PAH is an infrequent but severe vascular disorder with increased pulmonary arterial pressure (PAP) as an outcome of vascular remodeling. PAH is categorized by an increased mean PAP (of ≥ 25 mmHg at rest), increased pulmonary vascular resistance (PVR) of > 3 Wood units, and a pulmonary capillary wedge pressure (PAWP) ≤ 15 mmHg, given all the gold standard measurement is a right heart catheterization (RHC). Nonetheless, echocardiogram can additionally give an assessment of the severity of the disease assessing right ventricular pressure and estimating secondary signs of right ventricle strain [28]. PAH is connected with various aspects and conditions. Certain drugs have been linked with the development of PAH, including methamphetamines and anorexigens. Additionally, portal hypertension, connective tissue disease, and human immunodeficiency virus can result in PAH.

Heritable PAH (HPAH) diagnosis is pertinent when numerous genetic mutations are discovered. Bone morphogenetic protein receptor 2 (BMPR2) mutations are found in 75% of patients in this category [29–31]. The remaining 25% of patients are additionally connected with other genetic mutations as well. These include* BMP9*, SMAD3,* ENG*, and* ACVLR1*, which are also connected with HHT [32, 33]. These can all be found to encode for proteins that play a function in the transforming factor-beta (TGF-*β*) signaling pathway. Furthermore, BMPR2 deficiency has been previously seen to disrupt pulmonary vascular changes causing various defects in both systemic and heart circulation [28]. BMP signaling is tied into the pathogenesis of atherosclerosis and* BMPR2* mutations appear to produce unique singular fibrovascular lesions, affecting larger bronchial arteries. This could explain why increased quantities of hemoptysis are seen in PAH with* BMPR2 *mutations [28–30].

### 2.2. Hereditary Hemorrhagic Telangiectasia (HHT)

HHT is an autosomal dominant inherited disease that affects 1 in 5000 individuals worldwide. Arteriovenous malformations (AVMs) and mucocutaneous telangiectasia are outcomes of vascular dysplasia. AVMs can hypothetically cultivate in every organ; however, most affected organs are the liver, brain, and lung. These AVMs are vulnerable to hemorrhage and rupture, which leads to major morbidity and mortality. Pulmonary AVMs result in blood flow from the pulmonary artery to the pulmonary vein, resulting in a decreased filtering volume of the pulmonary capillary bed. Complications from pulmonary AVMs therefore include paradoxal emboli and hypoxemia. To screen for pulmonary AVMs in HHT patients, a contrast echocardiogram can be used [34]. In order to decrease the risk of these severe problems, patients should be treated with an endovascular intervention that occludes the feeding of the pulmonary AVM with vascular plugs or coils also known as an embolization [35]. Vascular malformation is existent in 32-78% of HHT patients. This occurs in three various types including hepatic artery to hepatic vein, portal vein to hepatic vein, and/or shunting from hepatic artery to portal vein [36, 37]. Additionally, these various AVMs can be directly involved in the vascular defects and impaired angiogenesis observed in HHT, which can be essentially elucidated by the breakdown of the endothelial cells [35–37].

**Figure 1:**
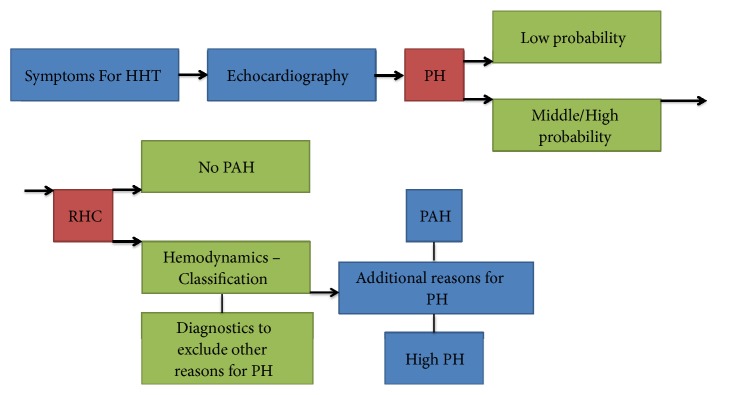
Flow chart of PH diagnosis in HHT. Abbreviations: HHT: hereditary hemorrhagic telangiectasia; PH: pulmonary hypertension; RHC: right heart catherization; PAH: pulmonary arterial hypertension.

The most essential clinical feature of HHT is epistaxis, from which 96% of patients suffer with HHT and additionally more than 50% before the age of 20 years old [38, 39]. A majority of cases are caused by mutations in the* ACVRL1* or* ENG* genes. These mutations result in decreased levels of functional proteins of ALK1 and Endoglin due to haploinsufficiency [40]. Another disease-triggering mutation has been found in the* SMAD4* gene, which results in a grouping of HHT and juvenile polyposis syndrome [41]. However, this mutation is rarer compared with others and only found in 1-2% of HHT patients. Most families associated with HHT have a distinctive mutation and more than 900 mutations are defined [42]. Mutations that cause HHT type 1 such as* ENG* are characterized by a higher frequency of cerebral and pulmonary AVMs, epistaxis, and mucocutaneous telangiectasia compared to* ACVRL1* mutations or HHT type 2. Most indications with HHT are progressive with age. Clinical signs are not only in age and subtype but also variable in severity between families with like mutations [43]. Genetic modifiers and etiological influences are considered to elucidate this clinical inconsistency [44]. Distinguishing between common symptoms of HHT and HHT complicated by PAH can be a perplexing endeavor. HHT patients frequently experience shortness of breath, exhaustion, and exercise intolerance due to hypoxemia and anemia resultant of pulmonary AVMs, psychological strain of a chronic disease, and epistaxis. Diagnostic management of PH in HHT is contingent on the existence of the symptoms as seen in Figure 1. If a patients' physical exam or history indicates PH, an echocardiogram should be implemented to measure the likelihood of PH.

### 2.3. Molecular Mechanisms of PAH and HHT

**Figure 2:**
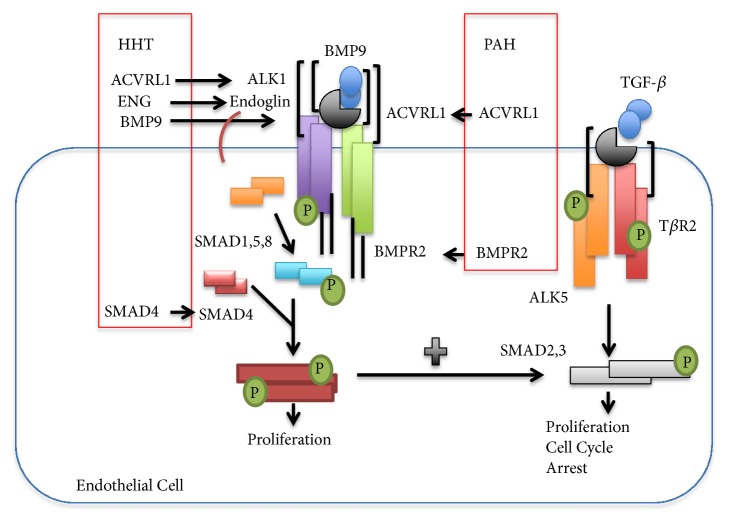
Diagram of the BMP9 and TGF-*β* pathway and involved genes and proteins in PAH and HHT. Two pathways are displayed: ALK1/SMAD1-5 and ALK5/SMAD2-3. Abbreviations: P: phosphorylation.

Signaling pathway families such as TGF-*β* have been considered to play an essential role in various cellular activities such as apoptosis, proliferation, and migration [45]. A complex pathway, TGF-*β*, plays an important part in the process of angiogenesis using two signaling pathways: ALK1-Smad 1/5/8 pathway and activin receptor-like kinase 5 (ALK5)-Smad2/3 pathway as demonstrated in Figure 2 [13, 14]. When vessels are produced, ECs migrate and proliferate. Once the wall of the capillary is developed, pericytes aid in stabilizing the vessel and inhibit endothelial migration and proliferation. This ultimately leads to vascular maturation, in which ALK5 plays significant role. Upregulation of Endoglin via ALK1 is a necessary receptor in the TGF-*β*/BMP signaling pathway, which is specifically expressed on proliferating ECs. Additionally, it has been established that endoglin counterbalances the steadying function of ALK5 [46, 47].* ACVRL1* and* ENG* mutations disturb TGF-*β*/BMP signaling, thus modifying pericyte recruitment and EC tubulogenesis triggering irregular capillary maturation and formation leading to vascular hyperbranching, AVMs, and venous expansion elucidating the irregular morphogenesis of vascular in HHT [13, 48].

Vascular function is also regulated by ECs through mediating the production of vasodilators, vasoconstrictors, and both inhibition and activation of SMCs. Outcomes due to inhibition of apoptosis of SMCs can lead to vascular remodeling and proliferation, eventually causing PAH. This outcome can be due to the disruption of BMP signaling and the SMAD1,5,8 pathway, as a consequence of an* ACVRL1* and* BMPR2* mutation [49–51]. However, both HHT and PAH originate in deficiencies in the ALK1/BMP9/Endoglin pathway. A signaling complex between ALK1 and BMPR2 is formed, which responds to BMP9 through binding with high affinity to Endoglin and ALK1 [32, 52]. Studies have indicated that BMP9 has been used in animal studies to treat PAH by stimulating* BMPR2* signaling. Additionally, the mutation of BMP9 can lead to syndrome with similar characteristics with [53–55]. Based on these lines of evidence, it may be plausible that BMP9 may have therapeutic benefits on HHT.

Difficulties of HHT can be furthered by heritable pulmonary arterial hypertension (HPAH); however, this is a rare case. Various mutations such as* ACVRL1* have been explained in HPAH patients but there seems to be a predominance of mutations in the nonactivating nondownregulating box (NANDOR) [42]. Nevertheless, most family members of HHT patients with HPAH will not develop HPAH, which suggests that further environmental or genetic influences are needed to develop HPAH characteristics [56]. Information of this disease grouping of PAH and HHT is significant while this particular grouping frequently leads to worse results than PAH alone [56]. A study by Li et al. compared HHT-PAH patients to IPAH patients, assessing their prognosis. One- and three-year survival rates were individually 78% and 53% for HHT-PAH patients, suggestively lower than patients with IPAH one- and three-year survival rates at 91% and 74% individually [56].* SMAD4* in the pathogenesis of HPAH is not completely known. While there are no HHT connected* SMAD4* mutation carriers defined with HPAH, there are two PAH patients in whom a mutation in the* SMAD4* gene is established [57]. Both diseases share differences between men and women. Epidemiological data demonstrates female predominance in several types of PAH and life expectancy with HHT produced by an* ENG *mutation [58, 59]. It is assumed that female hormones play an essential role in both diseases; nonetheless, the particular mechanisms are not yet completely comprehended [60–62].

### 2.4. Influence of ID3 on PAH and HHT

ID proteins are downstream targets of the* BMP* signaling pathway. While literature evidence is limited, influence of ID3 on both PAH an HHT has been demonstrated as seen in Table 1 through pathways previously highlighted in this review paper. Mutations in the* BMPR2* gene, encoding the type II BMP receptor, have been previously identified in patients with PAH, implicating BMP signaling in PAH. BMP receptors Ib and II together with* SMAD* 4,5,6, and 8 were downregulated in lungs but not kidneys of monocrotaline (MCT) rats. Expression of BMP/SMAD target genes* ID1* &* ID3* and* SMAD1* phosphorylation was decreased. Induction of the BMP/SMAD-responsive component of the* ID1 *promoter,* SMAD* expression, and* SMAD1* phosphorylation demonstrated a decrease in pulmonary arterial smooth muscle cells (PASMCs) from MCT-inoculated rats. Due to impaired BMP/SMAD signaling, the PASMCs from the MCT-inoculated rats were defiant to apoptosis stimulated by* BMP7* and* BMP4*. Based on this information, the results show how* ID3* is involved in BMP signaling, which transpires in the pathogenesis of human PAH [63]. Furthermore, Lowery et al. evaluated pulmonary expression of ID proteins in a mouse model of a hypoxia-induced PH. Results showed selective induction of* ID1* and* ID3 *expression in hypoxic VSMCs in vivo. Additionally, expression of* ID1* and* ID3* are increased by hypoxia in cultured pulmonary VSMCs in BMP-dependent nature. PH response to chronic hypoxia is dim between wild type and* id1* null mice. This is connected with a compensatory rise in* ID3* but not* ID2* expression in pulmonary VSMCs of* id1* null mice. Taken together, these findings indicate that expression of* ID1* and* ID3 *is regulated in a BMP-dependent fashion in hypoxic pulmonary VSMCs.* ID1* and* ID3* may also play compliant function in regulating BMP-contingent VSMC response to chronic hypoxia [64].

Yang et al. investigated ID protein expression in human PASMCs by immunoblotting and real-time PCR.* ID3* expression in pulmonary vessels was investigated in BMPR-II mutant mice and in patients with heritable PAH.* BMP4 *and BMP induced mRNA expression of* ID1, ID2*, and* ID3*. The BMP-stimulated initiation of* ID1* and* ID3* was evidently decreased in BMPR-II mutant PASMCs and in control PASMCs succeeding siRNA suppression of BMPR-II. Pulmonary arteries in* bmpr2* mutant mice and patients with heritable PAH exhibited decreased levels of* ID3* compared with control subjects. Furthermore, lentiviral* ID3 *overexpression reduced cell cycle progression and inhibited proliferation of PASMCs. Based on these lines of evidence,* ID3* is one of the ID proteins considered as critical downstream effector of BMP signaling in PASMCs. Both* ID1 *and* ID3* regulate proliferation of PASMCs via cell cycle inhibition, which may be intensified by inflammatory stimuli [9]. Gender also plays a large role in PAH development. Women develop PAH more frequently than men. Female non-PAH heritable PASMCs presented decreased messenger RNA and protein expression of* BMPR2*,* SMAD1*,* ID1, *and* ID3*. Phospho-SMAD1,5,8 and ID protein induction by* BMP4* was also decreased in female heritable PASMCs. In male heritable PASMCs, estrogen decreased messenger RNA and expression. The estrogen metabolite 4-hydroxyestradiol decreased phospho-SMAD1,5,8 and ID expression in female heritable PASMCs while increasing these in males corresponding with a decreased proliferative effect in male heritable PASMCs [10].

**Figure 3:**
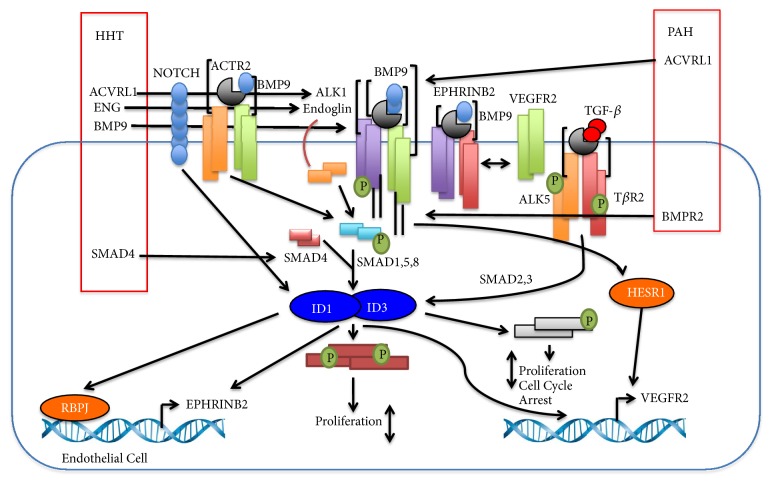
Diagram of how transcriptional regulator ID3 affects various pathways in HHT and PAH. Pathways included are ALK1/SMAD1-5, ALK/SMAD2-3, NOTCH, EPHRINB2–VEGFR2, and ALK1/ACTR2 via BMP or TGF-*β* signaling.

*ID3* has also been shown to transcriptionally reprogram lung endothelial cells via induction of proliferation and environmental toxicant PCB153. Committed ectopic* ID3* expression in lung endothelial cells contributed to endothelial-mesenchymal transition, cell migration, and cell proliferation. Using an established method to measure aberrant hyperproliferation of endothelial cells in PAH patients, Doke showed that established ectopic expression of* ID3* increased the size and number of vascular spheres. Using ChIP-Sequence and RNA-Sequence methodology,* ID3* is shown to be a part of a more general mechanism of transcriptional regulation. The ChIP-Sequence data showed that an important preference of* ID3* binding to motifs connected with transcription factors: BC11A, PRDM1, SMAD4, FOXJ3, IRF4 ZBTB6, GATA1, IRF1, and STAT2 [65]. Additionally both PAH and HHT have been understood to be involved with* ID3 *in a vascular remodeling and dysfunction capacity. Gene expression and machine learning analysis exhibited* ID3* as candidate target genes in PAH and HHT tissue and blood samples.* ID3* and candidate target genes including* ABCB6, ACP1, BYSL, CAD, CDH15, DCBLD2, DHRS3, DNMT1, ID3, MCM4*, and* NDUFA7 *were shown to be essential to various vascular remodeling pathways such as focal adhesion, oxidative phosphorylation, and cell cycle [66]. Lastly, using an angiogenesis model, Kim et al. found that* ALK1* ligand* BMP9 *induces* EPHRINB2* in an* in vitro* model of HHT type 2.* BMP9* stimulates both* ID1 *and* ID3*, which are both essential for full initiation of* EPHRINB2*. Loss of EPHRINB2 and* ALK1 *caused an increased arterial-venous anastomosis, while loss of* ALK1* not EPHRINB2 demonstrates an increased expression of VEGFR2 and development. It is also shown that BMP9 blocks EC development and is reliant on* BMPRII/ACTRII, ID1/ID3*, and* ALK1*. Taken together, loss of* ALK1* blocks BMP9 signaling which results in decreased expression of* EPHRINB2*, misregulated EC development and anastomosis, and heightened VEGFR2 expression [8]. Based on the scientific evidence that links* ID3* with both PAH and HHT, a schematic representation can be understood of how* ID3* can influence these disease outcomes from a mechanistic approach as summarized in Figure 3. Additional research is necessary in order to completely reveal how these molecular communications between* ID3* and PAH/HHT pathways impact these disease results.

## 3. Conclusion

Based on the existing literature,* ID3 *has shown to be connected with both PAH and HHT. Various studies have reported individual associations between* ID3*-PAH and* ID3*-HHT, respectively; however, the combination of all three is rare but may have strong implications on their outcomes.* ID3* is a transcriptional regulator seen to be essential in the promotion of cell cycle progression, endothelial cell activation, and embryonic vasculogenesis. Both diseases can be a result of mutations affecting various signaling pathways such as TGF-*β* and BMP, vital for processes such as angiogenesis. Clinical indicators may not be specific but primary diagnosis is significant for the applicable treatment and prognosis. Thus, the overall understanding of the interactions between* ID3* and these diseases is critical to the healthcare and scientific community working with PAH or HHT patients.

## Figures and Tables

**Table 1:** ID3 and PAH & HHT described in the scientific literature.

ID3 Associated Study Title	Authors	Year	Disease
Dysregulated bone morphogenetic protein	Morty et al.	2007	PAH
signaling in monocrotaline-induced			
pulmonary arterial hypertension			

ID family protein expression and regulation	Lowery et al.	2010	PH
in hypoxic pulmonary hypertension			

BMP induces EphrinB2 expression in	Kim et al.	2012	HHT
endothelial cells through an			
Alk1-BMPRII/ActRII-ID1/ID3-dependent			
pathway: implications for hereditary			
hemorrhagic telangiectasia type II			

Id proteins are critical downstream	Yang et al.	2013	PAH
effectors of BMP signaling in human			
pulmonary arterial smooth muscle cells			

Sex affects bone morphogenetic	Mair et al.	2015	PAH
protein type II receptor signaling in			
pulmonary artery smooth muscle			
cells			

ID3 contributes to the acquisition of	Das et al.	2015	IPAH, PAH
molecular stem cell-like signature in			
microvascular endothelial cells: its			
implication for understanding microvascular			
diseases			

ID3, estrogenic chemicals, and the	Avecilla V.	2017	PAH, HHT
pathogenesis of tumor-like proliferative			
vascular lesions			

The role of ID3 and PCB153 in the	Doke MA.	2018	PAH
hyperproliferation and dysregulation of			
lung endothelial cells			
